# A Pilot, Prospective, Observational Study to Investigate the Value of NGS in Liquid Biopsies to Predict Tumor Response After Neoadjuvant Chemo-Radiotherapy in Patients With Locally Advanced Rectal Cancer: The LiBReCa Study

**DOI:** 10.3389/fonc.2022.900945

**Published:** 2022-06-28

**Authors:** Raffaello Roesel, Samantha Epistolio, Francesca Molinari, Piercarlo Saletti, Sara De Dosso, Mariacarla Valli, Alessandra Franzetti-Pellanda, Letizia Deantonio, Maira Biggiogero, Paolo Spina, Sotirios Georgios Popeskou, Alessandra Cristaudi, Francesco Mongelli, Luca Mazzucchelli, Federico Mattia Stefanini, Milo Frattini, Dimitri Christoforidis

**Affiliations:** ^1^ Department of Visceral Surgery, Regional Hospital of Lugano, Ente Ospedaliero Cantonale, Lugano, Switzerland; ^2^ Istituto Cantonale di Patologia, Ente Ospedaliero Cantonale, Locarno, Switzerland; ^3^ Clinical Research Unit, Clinica Luganese Moncucco, Lugano, Switzerland; ^4^ Medical Oncology Clinic, Clinica Luganese Moncucco, Lugano, Switzerland; ^5^ Faculty of Biomedical Sciences, University of Southern Switzerland, Lugano, Switzerland; ^6^ Istituto Oncologico della Svizzera Italiana, Ente Ospedaliero Cantonale, Bellinzona, Switzerland; ^7^ Radiation Oncology Clinic, Oncology Institute of Southern Switzerland, Ente Ospedaliero Cantonale, Bellinzona, Switzerland; ^8^ Service of Radiotherapy, Clinica Luganese Moncucco, Lugano, Switzerland; ^9^ Department of Health Sciences, University of Eastern Piedmont, Novara, Italy; ^10^ Faculty of Science and Technology-ESP, University of Milan, Milano, Italy; ^11^ Department of Visceral Surgery, Lausanne University Hospital, Lausanne, Switzerland

**Keywords:** liquid biopsy, circulating tumor DNA (ctDNA), rectal cancer (RC), next-generation sequencing, watch and wait approach, LARC

## Abstract

**Introduction:**

Circulating tumor DNA (ctDNA) correlates with the response to therapy in different types of cancer. However, in patients with locally advanced rectal cancer (LARC), little is known about how ctDNA levels change with neoadjuvant chemoradiation (Na-ChRT) and how they correlate with treatment response. This work aimed to explore the value of serial liquid biopsies in monitoring response after Na-ChRT with the hypothesis that this could become a reliable biomarker to identify patients with a complete response, candidates for non-operative management.

**Materials and Methods:**

Twenty-five consecutive LARC patients undergoing long-term Na-ChRT therapy were included. Applying next-generation sequencing (NGS), we characterized DNA extracted from formalin-fixed paraffin embedded diagnostic biopsy and resection tissue and plasma ctDNA collected at the following time points: the first and last days of radiotherapy (T_0_, T_end_), at 4 (T_4_), 7 (T_7_) weeks after radiotherapy, on the day of surgery (T_op_), and 3–7 days after surgery (T_post-op_). On the day of surgery, a mesenteric vein sample was also collected (T_IMV_). The relationship between the ctDNA at those time-points and the tumor regression grade (TRG) of the surgical specimen was statistically explored.

**Results:**

We found no association between the disappearance of ctDNA mutations in plasma samples and pathological complete response (TRG1) as ctDNA was undetectable in the majority of patients from Tend on. However, we observed that the poor (TRG 4) response to Na-ChRT was significantly associated with a positive liquid biopsy at the T_op_.

**Conclusions:**

ctDNA evaluation by NGS technology may identify LARC patients with poor response to Na-ChRT. In contrast, this technique does not seem useful for identifying patients prone to developing a complete response.

## Introduction

Patients with locally advanced rectal cancer (LARC) are traditionally treated with neo-adjuvant chemoradiation therapy (Na-ChRT) followed by surgery with total mesorectal excision (TME) (1). In 15–20% of cases, Na-ChRT ultimately results in a pathological complete response (pCR), with no viable tumor in the final surgical specimen (2). The benefits of surgical resection in such cases have been questioned. Indeed, following the pioneering work of Habr-Gamma et al. (3), in patients with clinical, endoscopic, and radiologic complete response, non-operative management (NOM) is increasingly proposed (2, 4), with apparently equivalent oncological outcomes but significantly less treatment morbidity and fewer long-term functional problems (5, 6). However, because the accuracy of clinical and radiological re-staging after Na-CHRT remains suboptimal and the rate of local regrowth in NOM patients is still significant, ranging from 5 to 60% (5, 7–10), new reliable markers of pCR are needed.

The ability to detect in the bloodstream DNA fragments from tumor cells exhibiting tumor-specific genetic alterations has recently offered a new perspective for oncological staging and prognostic stratification (11). Circulating tumor DNA (ctDNA) derives from tumor cells undergoing apoptosis or necrosis, which release DNA into the blood stream (12, 13). In plasma samples, the mutations can be detected only in the presence of a cancer (primary or recurrent) and circulating tumor DNA (ctDNA) levels are related to cancer size (11). The value of such “liquid tumor biopsy” has been explored in different types of cancer (4), showing that the variation of ctDNA, both in terms of detection of specific mutations and frequency of mutation, is strictly correlated with the course of the disease. In particular, the presence and quantity of ctDNA has been associated with tumor staging and prognosis (11) and, as a consequence, the evaluation of liquid biopsies has been successfully introduced to monitor tumor burden and therapy resistance, to evaluate the presence of residual disease and to monitor recurrence (14). Indeed, it has been demonstrated that ctDNA decreases significantly in response to successful treatment with many types of chemotherapy, including immune checkpoint inhibitors (15, 16).

The value of liquid biopsies (4) is still unclear in rectal cancer, as only a few reports have been published concerning their clinical role (8, 14, 17). In particular, these publications reported that liquid biopsy could not be sensitive enough for detecting microscopic residual disease. However, these biomarkers can be used to monitor treatment response to Na-ChRT to detect disease recurrence, precede increases in CEA levels and radiological diagnosis, and to evaluate the effect of Na-CRT in patients with rectal cancer. Our hypothesis was that ctDNA could be used as a marker of tumor response to Na-ChRT, and identify patients with a complete response who could then undergo NOM. This study aimed to deepen the data reported in the literature and to explore the value of serial molecular analysis of ctDNA along the LARC treatment pathway as a novel blood-based biomarker of response to multimodal therapy.

## Material and Methods

### Study Design and Participants

This is a prospective study (ClinicalTrials.gov Identifier: NCT03699410) on patients treated for LARC in two hospitals in Southern Switzerland, the Regional Hospital of Lugano and the Clinica Luganese di Moncucco. Molecular analysis of all patients was performed at the Istituto Cantonale di Patologia EOC.

The recruitment of patients occurred from November 2018 to May 2020. Inclusion criteria were age >18 years, biopsy proven rectal adenocarcinoma, a clinical staging of a LARC, and candidate for a multimodal treatment strategy with long-course 5-fluorouracil based chemotherapy (5-FU) plus 50.4 Gy radiotherapy and TME surgery after the specific oncological multi-disciplinary meeting. Patients were excluded in the presence of contraindications to multimodal treatment. All patients were staged with a digital rectal examination, colonoscopy, contrast-enhanced thoraco-abdominal CT-scan, and pelvic MRI. Endorectal ultrasound was also performed when possible.

Serial blood sampling was performed at 7 different time-points along the treatment pathway: on the first and last days of radiotherapy (T_0_ and T_end_ respectively), at 4 (T_4_) and 7 (T_7_) weeks after radiotherapy, on the day of surgery (10 weeks after radiotherapy) (T_op_), and at 3–7 days after surgery (T_post-op_). During surgery, a blood sample from the inferior mesenteric vein was collected when technically feasible (T_IMV_). This study was approved by the local ethical committee (Ref. number: 2018-01193 I CE 3378), and all patients included provided written informed consent. Clinical patient data were collected from the electronic charts.

**Figure 1:**
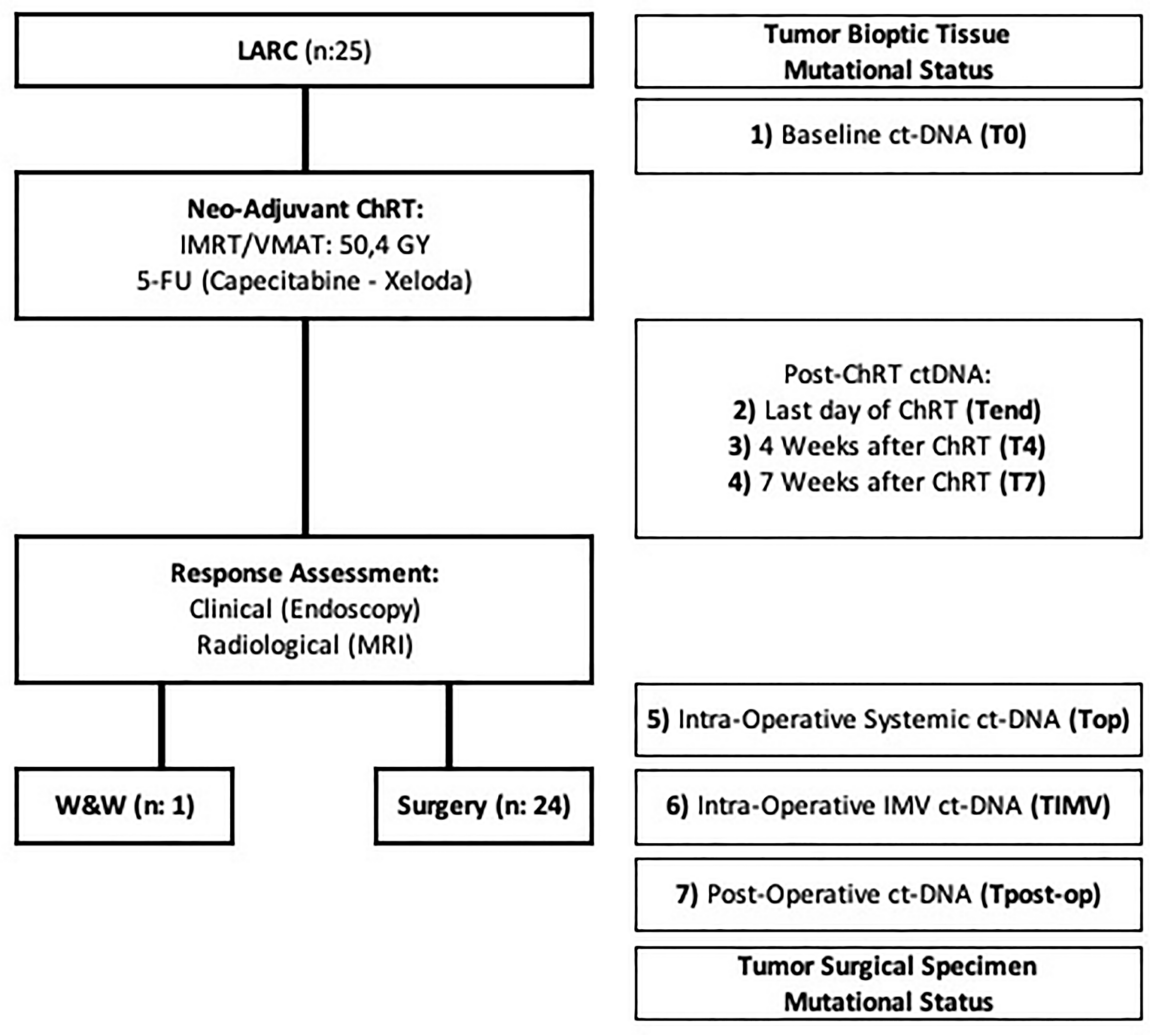
Flow chart describing the study development. ChRT, chemoradiation therapy; ctDNA, circulating tumor DNA; IMRT, intensity-modulated radiation therapy; LARC, locally advanced rectal cancer; MRI, magnetic resonance imaging; VMAT, volumetric modulated arc therapy; W&W, watch and wait approach; 5-FU, 5-FluorUracil.

Twenty-five consecutive patients who completed the Na-ChRT were prospectively included between November 2018 and May 2020. After proper re-assessment with clinical and radiological evaluation with CT scan and pelvic MRI at 7 weeks post Na-ChRT, 24 of them underwent TME surgery, whereas one patient with a clinical complete response was treated with a “watch and wait” (W&W) approach (**Figure 1**).

### Histopathology

The resected specimens were evaluated by an expert pathologist of the gastrointestinal tract. In addition to standard histopathological analysis (yTN stage, margins, TME quality, differentiation grade, lymphovascular and perineural invasion, budding), cancers were specifically classified on the basis of the tumor regression grade (TRG), according to Mandard et al. (18, 19).

### Tissue Mutational Analyses

Genomic DNA was extracted from three 8 µm-thick serial sections of the selected formalin-fixed paraffin-embedded (FFPE) tissue block corresponding to the tumor biopsy (obtained before any treatment) and to the final surgical specimen (collected after Na-ChRT). DNA was extracted using the QIAamp DNA FFPE tissue kit (Qiagen, Chatsworth, CA, USA), following the instructions of the manufacturer.

The molecular characterization was performed in a qualitative and semi-quantitative manner using a next-generation sequencing (NGS) approach on the S5 Ion Torrent (IOT) platform, by applying the Ion AmpliSeq™ Cancer Hotspot Panel v2 (CHPv2) (ThermoFisher Scientific, Waltham, MA, USA). The CHPv2 panel provides data about the mutational status of 50 genes, including the most relevant and frequently mutated genes in rectal cancer (i.e., KRAS, NRAS, APC, TP53, PIK3CA, BRAF, and STK11). The extracted DNA was quantified by a QUBIT^®^ (ThermoFisher Scientific) fluorometer and 10 ng of DNA was used for library preparation. Then the samples were quantified by real-time PCR and diluted with sterile water to achieve a final concentration of 100 pM for further NGS processing.

IOT sequencing data were defined as evaluable only if the coverage analysis of every sample was acceptable (i.e., the target regions were covered at least by 500 reads), if the mean depth values were greater than 1,100× and if the uniformity was greater than 90%.

For precise and reliable characterization of variants, the limit of detection (LOD) for single nucleotide variant identification was set at 2–5%. All the mutations known in literature as being benign variants/polymorphisms and all the variants included in the intronic regions (with the exception of UTR or splice site regions) were excluded.

### Plasma Mutational Analyses

At each different collection time-point, 20 ml of blood was drawn in Cell-Free DNA (cfDNA) BCT^®^ tubes (Streck; Omaha, NE, USA). After centrifugation, the plasma was stored at −80°C until DNA extraction and molecular characterization. The extraction of cfDNA was made starting from 4 ml of plasma using the MagMax™ cfDNA isolation kit (ThermoFisher Scientific), according to the instructions of the manufacturer. The ctDNA was quantified by the QUBIT^®^ fluorometer (ThermoFisher Scientific), and 50 ng of ctDNA were used for library preparation. Then the samples were quantified by real-time PCR and diluted with sterile water to achieve a final concentration of 100 pM for further NGS processing.

The analysis of molecular alterations in plasma was performed using the Oncomine™ colon cfDNA assay (ThermoFisher Scientific) on the S5 IOT platform. This panel provides data about the mutational status of 14 genes and 46 target regions, including KRAS, NRAS, APC, TP53, PIK3CA, and BRAF genes.

To achieve the sensitivity required for this methodology in plasma analyses (LOD = 0.1%), we verified from coverage analyses that the samples had a median read coverage value of ≥20,000 and a median molecular coverage value of >2,500. To call variants, we used software and parameters provided by ThermoFisher Scientific through Torrent Suite software, which are specific for this analysis. All the mutations under the LOD, or those known in the literature as being polymorphic or included in intronic regions, were excluded.

### Statistical Analysis

Statistics were performed using R, version 4.0.3. Continuous variables were expressed as the median and interquartile range (IQR); discrete variables were expressed as frequent counts and relative percentages. Fisher’s exact test was used to compare the proportion of patients with plasma mutations at baseline, the day of surgery, and post-operative. All p-values were two-sided; values with p <0.05 were considered statistically significant.

## Results

All the clinicopathological characteristics of these patients and their correlation to ctDNA values are summarized in **Table 1**. The median age was 65 years (IQR: 59–70). Twenty-two patients (88%) were men. The median distance of the tumor from the anal verge was 6 cm (IQR: 3–7). A pathological complete response (TRG1) was observed in 4 cases (**Table 1**).

**Table 1:** Clinicopathological characteristics of the study cohort.

		ct-DNA: Baseline			ct-DNA: post-ChRT (pre-OP)			ct-DNA: post-OP		
		Pos. (+) n: 20	Neg (–). n: 3	*p*	Pos. (+) n: 7	Neg. (-) n: 17	*p*	Pos. (+) n: 5	Neg. (-) n: 18	*p*
**Age (years):**										
Median	**65**	64	66	0,96	67	65	0,56	60	67	0,71
IQR	**59-70**									
**Gender, n. (%):**										
Male	**22 (88%)**	19	1	1	6	15	0,01*	5	15	0,01*
Female	**3 (12%)**	1	2		1	2		0	3	
**Dist. a.v. (cm), n. (%):**										
> 4	**17 (68%)**	15	1	0,20	5	12	1	4	12	1
≤ 4	**8 (32%)**	5	2		2	5		1	6	
**MRI Clinical T, n. (%):**										
cT1-2	**4 (16%)**	4	0	0,97	0	4	0,28	0	4	0,53
cT3-4	**21 (84%)**	16	3		7	13		5	14	
**MRI Clinical N, n. (%):**										
cN0	**2 (8%)**	2	0	1	1	1	0,51	1	1	0,40
cN1-2	**23 (92%)**	18	3		6	16		4	17	
**Pre-treat. CEA, n. (%):**										
Normal (< 5 ng/ml)	**15 (60%)**	12	2	1	4	11	0,61	3	11	0,61
High (≥ 5 ng/ml)	**6 (25%)**	4	1		2	3		2	4	
N.A.	**4 (16%)**									
**End ChRT - Surg (days)**										
Median	**70**	69,1	71,5	0,65	67,5	70	0,511	71	68,7	0,55
IQR	**67-72**									
**Clinical-CR, n. (%):**										
No	**19 (76%)**	17	2	0,45	7	12	0,27	4	14	1
Yes	**6 (24%)**	3	1		0	5		1	4	
**MRI-CR, n. (%):**										
No	**18 (72%)**	15	3	1	7	11	0,13	4	13	1
Yes	**7 (28%)**	5	0		0	6		1	5	
**Res. Margin, n. (%):**										
R0	**22 (92%)**	18	3	1	6	16	0,51	4	17	0,34
R1	**2 (8%)**	2	0		1	1		1	1	
**Pathological T, n. (%)**										
ypT0-2	**10 (42%)**	7	2	0,54	2	8	0,65	1	8	0,61
ypT3-4	**14 (58%)**	13	1		5	9		4	10	
**Pathological N, n. (%)**										
ypN0	**18 (75%)**	16	2	0,54	5	13	1	3	14	0,57
ypN1-2	**6 (25)**	4	1		2	4		2	4	
**LnVi, n. (%)**										
Negative	**17 (71%)**	13	3	0,53	5	12	1	3	13	0,62
Positive	**7 (29%)**	7	0		2	5		2	5	
**Pni, n. (%):**										
Negative	**19 (79%)**	15	3	1	6	13	1	4	14	1
Positive	**5 (21%)**	5	0		1	4		1	4	
**Tumor Budding, n. (%):**										
≥ 30	**11 (46%)**	11	1	0,59	3	8	1	3	8	1
< 30	**13 (54%)**	9	2		4	9		2	10	
**Tumor Grading, n. (%):**										
G2	**18 (90%)**	15	2	1	5	13	0,52	3	14	0,29
G3	**2 (10%)**	2	0		1	1		1	1	
G N.A. (pCR)	**5**									
**pCR (ypT0N0), n. (%):**										
No	**20 (83%)**	17	2	0,45	6	14	1	4	15	1
Yes	**4 (17%)**	3	1		1	3		1	3	
**Recurrence, n (%):**										
Yes	**5 (21%)**	4	1	0,54	2	3	0,61	2	3	0,29
No	**19 (79%)**	16	2		5	14		3	15	

Fisher’s exact test was performed excluding the patients followed up by watch-and-wait approach. *indicates a p-value of <0.05. a.v., anal verge; CEA, carcinoembryonic antigen; CRT, chemoradiation therapy; CR, complete response; ctDNA, circulating tumor DNA; LnVi, Lympho-vascular invasion; MRI, magnetic resonance imaging; N.A., not analysed; pCR, pathological complete response; Pni, Perineural invasion; Res, Resection Margin.

### Evaluation of the Mutational Landscape and Statistical Analyses

**Figure 2:**
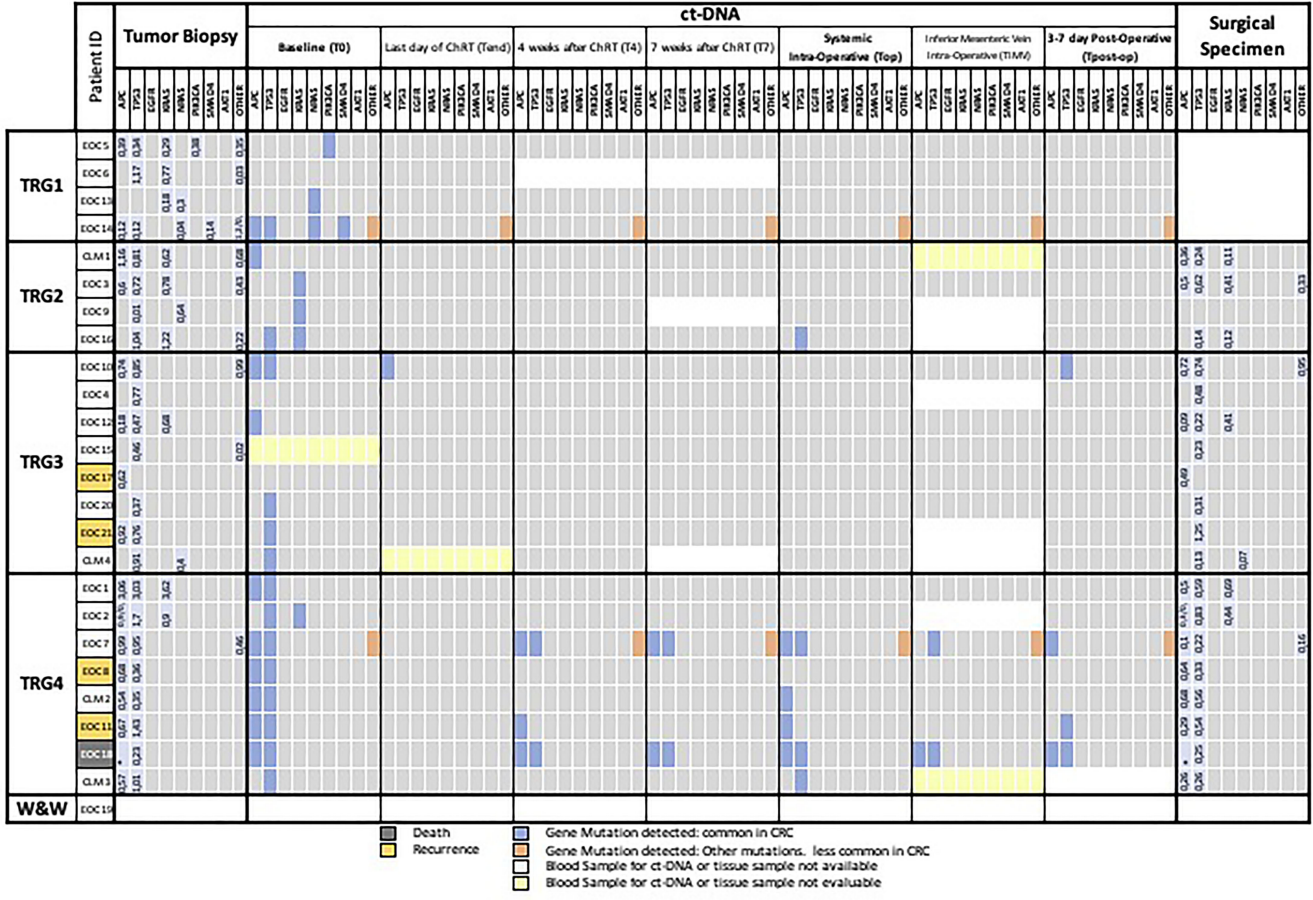
Mutational landscape of tumor biopsy, plasma samples, and surgical specimen. The meanings of the colors are shown in the legend below the figure. CRC, colorectal cancer; T0, plasma sample of the first day of radiotherapy; Tend, plasma sample of the last day of Na-ChRT, T4, plasma sample at 4 weeks after Na-ChRT; T7, plasma sample at 7 weeks after Na-ChRT; Top, plasma sample at day of surgery (10 weeks after Na-ChRT); T_IMV_, plasma sample from the inferior mesenteric vein at day of surgery (10 weeks after Na-ChRT); TRG, tumor regression grade; W&W, Watch and Wait approach.

In all patients, the tumor biopsy was characterized by at least one mutant gene. In more detail, we found an APC mutation in 15 cases, a TP53 mutation in 22 cases, a KRAS mutation in 9 cases, a NRAS mutation in 4 cases, a PIK3CA mutation in 1 case, a SMAD4 mutation in 1 case, and mutations in other genes in 9 cases (**Supplementary Tables 1–4**). Overall, 4 patients showed 1 mutation, 9 patients 2 mutations, 7 patients 3 mutations, 2 patients 4 mutations, and 2 patients 5 mutations. The same mutations found in the diagnostic biopsy were detected also in the resection specimen with the exception of one case that became totally wt (EOC9, TRG2), excluding, of course, patients with complete pathological response (**Figure 2**).

From 175 plasma samples planned (7 for each of the 25 patients enrolled), 157 were actually collected and included in the analyses. In 6 patients, the mesenteric vein sampling was unsuccessful for technical reasons, 5 peripheral blood samples were missed, and 7 samples from the patient who underwent the W&W approach were not collected. A mutational landscape table has been prepared as an overview of the findings (**Figure 2**).

Of the 157 plasma samples analyzed by NGS, 4 samples were not evaluable due to low coverage values (CLM1-T_IMV_, EOC15-T_0_, CLM4-T_end_, and CLM3-T_IMV_) (**Figure 2**).

At baseline (T_0_), one or more somatic mutations were detected in the plasma of 20 out of 23 evaluable patients (87%); the mutations were the same as those detected in the tissue biopsy. Three cases showed no mutations in baseline (T_0_) compared with preoperative biopsy (EOC6, EOC4, and EOC17) (**Figure 2**).

The mutations that more frequently presented a correspondence between biopsy and plasma sample at T0 were located in the APC (9/15, 60%) and TP53 (14/22, 63%) genes, whereas minor correspondence was found concerning KRAS (3/9, 33%) genes and non-common CRC genes (2/9, 22%) (**Figure 2**).

In the subsequent ctDNA analyses (T_4_, T_7_), we observed a further reduction in the positivity of the liquid biopsies regardless of the TRG of the patient. After 4 (T_4_) and 7 weeks (T_7_) at the end of the neo-adjuvant treatment, there were only 4 (EOC14 with TRG1 and EOC7, EOC11, and EOC18 with TRG4) and 3 positive samples (EOC14 with TRG1 and EOC7, EOC18 with TRG4), respectively (**Figure 2**).

On the day of surgery (T_op_), after a median interval at the end of the neo-adjuvant treatment of 114 days (IQR: 112–117), there were seven (7/24, 29%) patients with at least one mutant allele detectable in the systemic blood (**Figure 2**), five of which were TRG4, one was TRG2 and one TRG1. The patient with TRG1 (EOC14) presented a persistent mutation, uncommon in CRC, related to the GNAS gene (p.R201H) (**Figure 2**). At further analysis, this mutation was present in the tumor biopsy and in the surgical resected specimen but not in the healthy tissue of the patient. This patient (EOC14) was free of disease at last follow-up (17 months).

Intra-operative blood samples from the inferior mesenteric vein (T_IMV_) could not be taken or were not evaluable in 8/24 (33%) patients (EOC2, EOC4, EOC9, EOC16, EOC21, CLM1, CLM3, and CLM4) (**Figure 2**). The only two T_IMV_-positive patients were also Top positive (EOC7 and EOC18), but two other Top positive patients were T_IMV_-negative (EOC10 and EOC11), indicating a lower detection rate of ctDNA in mesenteric blood compared with systemic blood (**Figure 2**).

Mutational analysis of the final surgical specimens resulted positive in 19/20 cases (95%), with 1 case without any mutation and classified as TRG2, confirming persistence of the index mutations, not detected in Top ctDNA in most patients.

At T_post-op_, five patients (5/23, 22%) had detectable ctDNA (**Figure 2**). Of those, one was the previously discussed TRG1 case with GNAS mutation (EOC14), one patient had R1 resection of a T4 abscessed tumor, developed rapidly progressive lymphangitic carcinomatosis and died 2 months after surgery (EOC18); one patient had R0 resection for a TRG4 ypT4N0, LnVi(−), Pni(−), G2 tumor and presented liver metastases 12 months after surgery (EOC11). The last two patients (EOC10 and EOC7) presented TRG3, ypT3N0 G2 and TRG4, ypT3N1, G2 tumors, and were free of disease at the last follow-up at 17 and 20 months, respectively.

The overall median follow-up was 14 months (IQR: 8–14), and an additional three patients with negative liquid biopsies at T_post-op_ developed disease recurrence. One (EOC21) had loco-regional lymph node recurrence and two others had systemic recurrence (EOC17 in the liver and EOC8 in the lungs). No significant associations between baseline (T_0_), pre-operative (T_op_) and post-operative (T_post-op_) ctDNA status and any clinicopathological factor were found (**Table 1**). However, TRG 4 was significantly associated with the presence of mutations in liquid biopsy at T_op_ (p = 0.04).

## Discussion

An increasing number of studies are investigating the role of ctDNA in the management of patients affected by CRC, but only a few focused on the potential value of this marker as an indicator of response to pre-operative therapy in patients with LARC (20–24). We offered our contribution to this major clinical research need by investigating the kinetics of this innovative marker through sequential evaluations during the pre-operative therapy and a double tissue assessment of the initial biopsy and the final surgical specimen. We focused our attention on a methodology characterized by a LOD (described as about 0.1% from the website of the manufacturer), potentially enabling the identification of gene mutations and small indels, according to the experience in other cancer types, such as lung adenocarcinoma (25). The NGS approach uses commercially available kits and it is easy to implement in each laboratory of molecular pathology. In addition, compared to other standard assays, it has, firstly, the advantage of allowing one to characterize different genes (a feature particularly relevant to monitoring the rise of small clones with different molecular profiles) in a single run using a smaller quantity of DNA for obtaining a large amount of molecular data and, secondly, good sensitivity (0.1%) in comparison to some of the most common PCR-based methodologies.

In our case series, ctDNA detectability with NGS technology using barcoding was not an adequate molecular marker of the overall disease status. Several aspects lead us to think that the main reason lies in the sensitivity of the technology applied for the identification of circulating tumor mutations (NGS Sequencing methodology). First, the detectability of circulating DNA was almost complete at the time of diagnosis and only after the start of neo-adjuvant therapy was the “liquid biopsy signal” lost.

In all cases, we extracted a low quantity of ctDNA (**Supplementary Table 3**), indicating no problems in the pre-analytical phase (absence of cell lysis before plasma extraction). Moreover, since the mutations included in the panel adopted for ctDNA were expressed in 19/20 resected tumors (**Supplementary Table 4**), and since the percentage of the mutations detected in plasma was very low even in the baseline sample, we hypothesize that the absence of signal in the follow-up of the patients is related to false-negative results related to quantitative (insufficient mutant allele content) rather than a qualitative (lack of inclusion of altered genes in the NGS panel and/or a tumoral mutation switch) problem. This assumption is corroborated by the fact that, at the time of resection, the majority of patients still contain tumor cells (TRG >0), which should release mutant DNA; as we do not see the signal, we interpret it as insufficient sensitivity of the NGS approach. On the other hand, there are other situations (i.e., brain tumors and brain metastases) where it was clearly demonstrated that although in the presence of tumor cells, the ctDNA samples are negative (11, 26). Our data, therefore confirm that a consistent number of tumor cells (as at baseline or in TRG4 cases after Na-ChRT of our cohort) is a condition required for NGS to demonstrate the presence of mutations in ctDNA.

We had assumed that intra-operative liquid biopsies from the IMV might be more sensitive than systemic ones by avoiding a possible first-pass hepatic clearance effect. Interestingly, our data indicate the contrary, as in half of the Top positive patients, ctDNA was undetectable at T_IMV_. Given that Na-ChRT induced tumor shrinkage, it probably occurred that the number of replicating tumor cells releasing DNA mutated into the bloodstream was insufficient to be detected with an NGS approach. This is also supported by the significant association between tumors with poorer response to neoadjuvant therapy (TRG4) and liquid biopsy positivity at the time of surgery. Moreover, the only mutations that persisted at Top related to genes APC and TP53, both tumor suppressor genes that act through mechanisms of senescence and apoptosis that play a critical role in regulating DNA repair in response to radiation (27–29). During the comparison between the molecular data obtained for tissue biopsies and for plasma samples, we do not consider the fact that the two different panels, one used for tissue DNA and the other for ctDNA, provide data for a different number of genes (50 versus 14 genes) because all genes mutated in tissue biopsies were also included in the OncomineTM colon cfDNA assay (ThermoFisher Scientific). As a consequence, the absence of mutations in plasma compared to initial tissue specimens is not because these genes are represented differently in the two panels. On the other hand, there are now on the market larger panels for the characterization of liquid biopsies. However, the technology and, as a consequence, the sensitivity are the same, so we believe that the use of larger panels in an NGS context does not result in a more reliable method for assessing the presence of few cancer cells in LARC patients treated with Na-ChRT.

In our experience, the NGS sequencing technology was therefore not sensitive enough to be useful in cases with a regular or good response to Na-ChRT characterized by a low tumor burden. We were not able to assess a significant correlation between the detectability of ctDNA postoperatively and the prognosis. Since ctDNA half-life is estimated to be less than 2 h (30) and we performed the T_post-op_ sampling 3–5 days after oncologic resection of the primary rectal tumor (before any possible adjuvant therapy), we would have expected positive liquid biopsy in all patients who developed systemic recurrence. However, only 2/5 patients with a disease recurrence at follow-up had ctDNA detectable postoperatively, and notably, these were both TRG4 tumors, with R1 resection in one case. We were probably again unable to see the metastatic spread of the disease by ctDNA earlier than morphologically manifested due to lack of sensitivity. Curiously, as previously mentioned, one patient who experienced pCR presented a detectable p.R201H mutation in the GNAS gene during the treatment pathway and postoperatively. GNAS is an oncogene acting through the regulation of the cAMP production and it is found in CRC only in 2.5% of the cases (31), indeed GNAS mutations are more commonly associated with cystic pancreatic neoplasm (32). Notably, this mutation was not identified in the healthy tissue of the patient (at further analysis) and therefore should be considered tumor specific rather than germinal. The patient was free of disease at the last follow-up (17 months) and pancreatic involvement was not identified by pancreatic magnetic resonance imaging (MRI). However, a systemic late recurrence of rectal cancer [reported as frequent as 15% of cases in pCR at 4 years (33)] or a misdiagnosed pancreatic cystic neoplasm will be taken into account at further follow-up.

Few other studies have explored the value of ctDNA as a biomarker of response to NaCRT. Murahashi et al. (20) applied a similar methodology on 85 patients and taking 3 liquid biopsies (at baseline, post-neoadjuvant treatment, and postoperatively) with no analysis of the mutational status of tissues (biopsy and surgical specimen). Only 49 (58%) patients had a detectable mutation at baseline. They observed no significant association between ctDNA status and the rate of responders (pCR patients). However, they reported that a change in ctDNA status between baseline and post-treatment was an independent predictor of a bad response to neo-adjuvant treatment. This conclusion was strongly driven by findings in 3 patients who did not have positive liquid tumor biopsy at diagnosis and who were instead positive following neo-adjuvant therapy. These cases were probably those with low initial tumor burden but unfavorable tumor “biology” that presented disease-progression during Na-ChRT. In our cohort, we did not observe any cases with these features, so we cannot confirm or reject the conclusions of Murahashi and colleagues. Pazdirek et al. (34) explored the changes in plasma ctDNA detectability in 33 patients before and one week after the initiation of Na-ChRT for rectal cancer using denaturing capillary electrophoresis as the first step and a high-resolution “BEAMING assay” directed at the detection of KRAS-specific ctDNA in a subgroup of patients. Their goal was to identify non-responders who may not benefit from NaCRT. The authors observed a clear reduction in ctDNA levels in all patients during the initial week of NCRT but without any direct association with the objective clinical response evaluated by TRG or TNM. Similar to our work, a group from Shangai (35) performed serial liquid biopsies before, during, and after Na-CRT and after surgery in 103 patients, 89 of whom had detectable ctDNA mutations at baseline. They used next-generation panel sequencing with a reported depth of approximately 4,000×. They found a significant association between TRG, pCR, and the disappearance of ctDNA after Na-ChRT. They suggested that combining liquid biopsies with post Na-ChRT MRI could improve the detection accuracy of complete responders. Finally, in another multicenter Chinese study with a similar design using NGS on 78 patients with detectable mutations at baseline, ctDNA was cleared with Na-ChRT before surgery in all but 11 patients. No patients with pCR had detectable preoperative ctDNA. The baseline liquid biopsy also seemed important as the median variant allele frequency (VAF) of mutations in baseline ctDNA was a strong independent predictor of metastasis free survival (HR, 1.27; *P <*0.001) (36).

Liquid biopsies have been advocated for response assessment and relapse monitoring in solid tumors, a practice that may be encouraged by a new commercially available plasma test (37). Two of the main techniques for ctDNA analyses are the Polymerase Chain Reaction (PCR) and Next Generation Sequencing (NGS)-based. PCR [such as Droplet Digital PCR or beads, emulsion, amplification, magnetics (BEAMing) techniques] relies on the detection of specific known mutations using primers that are complementary to the mutant sequences and offers high levels of sensitivity but is limited to the detection of either a single or a small number of known mutations (38). Next Generation Sequencing (NGS) based techniques theoretically enable sequencing of the entire genome and are considered more comprehensive, assessing several dozens of genes simultaneously (39, 40). In addition, NGS is able to detect mutational allele frequencies of <1% (41). Each of these two methods are currently suitable for the research on ctDNA in colorectal cancer (42), but notably the more interesting results have been reported for tumors with a large disease burden as in metastatic setting (13) where the sensitivity can be moderate because the concentration of ctDNA is much larger. The main difference between NGS and PCR-based techniques (e.g., ddPCR), besides the aforementioned number of alterations and markers detected, is the fact that ddPCR is the preferred option only if one or a few specific mutations, detected in the primary tumor, need to be monitored in the follow-up of patients. ddPCR, as well as all other assays not based on an NGS approach, is not useful for assessing the identification of new mutations arising from small clones that may acquire clinical relevance, possibly reaching dominance. Recently, great progress has been made for ctDNA sequencing methods to detect extremely low levels of mutation frequency (25), as INtegration of VAriant Reads (INVAR) pipeline (43) and that could enhance the sensitivity of liquid biopsies for LARC. However, when commercially available, a cost-effectiveness balance should be taken into account before implementing these highly expensive assays, for which large-scale practical applications could be unrealistic.

The limitation of this study is potentially the small sample size. However, the gap in sensibility for ctDNA detection by the NGS technology would have hardly been compensated for with broader patient inclusion. The consistency of our results relies mostly on the accurate analysis in both a semi-quantitative and qualitative manner and a panel covering the mutational status of 14 genes (the most relevant in rectal cancer development) and 46 target regions. Moreover, we analyzed 7 liquid biopsy samples and two tissue samples (biopsy and final surgical specimen) per patient at precise moments along the treatment pathway to exclude timing as a confounding factor.

In conclusion, we observed that ctDNA status with NGS technology was not a good marker for defining LARC patients who are more prone to developing a complete response to Na-ChRT; however, persistence of ctDNA molecular alterations after this treatment could be predictive of poor-response. Future studies, implementing commercially available technology for ctDNA, should then focus on patients with positive pre-operative liquid biopsy. This subgroup of patients, with high systemic disease burden and/or poor tumoral regression, may benefit from additional pre-operative chemotherapy.

## Data Availability Statement

The datasets presented in this study can be found in online repositories. The names of the repository/repositories and accession number(s) can be found in the article/**Supplementary Material**.

## Ethics Statement

The studies involving human participants were reviewed and approved by the Swiss Ethics Haus der Akademien, Bern. The patients/participants provided their written informed consent to participate in this study.

## Author Contributions

MF, DC, and AFP designed the project. RR and SE wrote the first draft of the manuscript. RR, PiS, SD, MV, AFP, LD, MB, and DC selected the cohort for the analyses. PaS and LM selected all the specimens at histologic level. SE, FMol, and MF performed all the molecular characterization. SE, FMol, and MF evaluated IOT results. RR, FMS, and MF performed statistical analysis. RR, SE, MF, and DC prepared the final version of the manuscript. MF and DC supervised the whole project. All authors listed have made a substantial, direct, and intellectual contribution to the work and approved it for pubblication.

## Funding

This project has been funded by a Grant of “Fondazione San Salvatore” (Ticino-Switzerland) for the research against human cancer and Research fund of Clinica luganese Moncucco.

## Conflict of Interest

The authors declare that the research was conducted in the absence of any commercial or financial relationships that could be construed as a potential conflict of interest.

## Publisher’s Note

All claims expressed in this article are solely those of the authors and do not necessarily represent those of their affiliated organizations, or those of the publisher, the editors and the reviewers. Any product that may be evaluated in this article, or claim that may be made by its manufacturer, is not guaranteed or endorsed by the publisher.

## Abbreviations

cfDNA, cell-free DNA; ChRt, chemoradiation therapy; CRC, colorectal cancer; ctDNA, circulating tumor DNA; FFPE, formalin fixed paraffin embedded; INVAR, integration of variant reads; IOT, Ion Torrent^®^; IQR, Interquartile Range; LARC, locally advanced rectal cancer; LOD, limit of detection; MRI, magnetic resonance imaging; Na-ChRT, Neo-adjuvant chemoradiation therapy; NGS, next-generation sequencing; NOM, non-operative management; pCR, pathologic complete response; TME, total mesorectal excision; TRG, tumor regression grade; VAF, variant allele frequencies; W&W, watch and wait approach; 5-FU, 5-fluorouracil based chemotherapy.
