# Supplementary material for: A Pilot, Prospective, Observational Study to Investigate the Value of NGS in Liquid Biopsies to Predict Tumor Response After Neoadjuvant Chemo-Radiotherapy in Patients With Locally Advanced Rectal Cancer: The LiBReCa Study
Source: Front Oncol. 2022 Jun 28;12:900945. doi: 10.3389/fonc.2022.900945 (PMC9274270; doi:10.3389/fonc.2022.900945)
Supplement: Supplementary file 1 [file DataSheet_1.docx]

**Supplementary Table 1: Mutations detected in tumor biopsy tissue samples.**

| **TRG** | **Patient ID** | **Baseline TNM Stage** | **Mutations of Tumor Biopsy** | | | | | | | | |
| --- | --- | --- | --- | --- | --- | --- | --- | --- | --- | --- | --- |
|  |  |  | **APC** | **TP53** | **EGFR** | **KRAS** | **NRAS** | **PIK3CA** | **SMAD4** | **AKT1** | **Other genes** |
| 1 | **EOC 5** | T3 N+ | E1379* | R306* |  | G13D |  | E545K |  |  | FBXW7: R465C |
|  | **EOC 6** | T3 N+ |  | R282W |  | G13D |  |  |  |  | CDKN2A: T79I |
|  | **EOC 13** | T2 N+ |  |  |  | G12D | Q61R |  |  |  |  |
|  | **EOC 14** | T3 N+ | Y1376Cfs*9 | R282W |  |  | Q61L |  | R361S |  | ATM: F858L  + GNAS: R201H |
| 2 | **CLM 1** | T3 N+ | E1379* | V272M |  | G12V |  |  |  |  | FBXW7: D399Y |
|  | **EOC 3** | T2 N+ | P1453Lfs*20 | G244D |  | G13D |  |  |  |  | FBXW7: R505C |
|  | **EOC 9** | T3 N+ |  | Y220C |  |  | G12V |  |  |  |  |
|  | **EOC 16** | T4 N+ |  | R273H |  | G12V |  |  |  |  | ERBB4: H295Lfs*2 |
| 3 | **EOC 10** | T3 N+ | R1114* | R273C |  |  |  |  |  |  | MET: R988C |
|  | **EOC 4** | T3 N+ |  | V216M |  |  |  |  |  |  |  |
|  | **EOC 12** | T3 N+ | E1309Dfs* | Q100R |  | A146V |  |  |  |  |  |
|  | **EOC 15** | T3 N+ |  | R213* |  |  |  |  |  |  | KIT: P573L + ATM: F858L |
|  | **EOC 17** | T4 N+ | E1097* |  |  |  |  |  |  |  |  |
|  | **EOC 20** | T2 N+ |  | I195T |  |  |  |  |  |  |  |
|  | **EOC 21** | T3 N0 | K1310Rfs*11 | R248Q |  |  |  |  |  |  |  |
|  | **CLM 4** | T3 N+ |  | C238Y |  | G13D |  |  |  |  |  |
| 4 | **EOC 1** | T3 N+ | Q1378* | S241F |  | A146V |  |  |  |  |  |
|  | **EOC 2** | T3 N+ | R1114* + T1556Nfs*3 | Y220C |  | G12C |  |  |  |  |  |
|  | **EOC 7** | T4 N+ | Q1367* (49.7%) | R213* (47.6%) |  |  |  |  |  |  | FBXW7: S582L |
|  | **EOC 8** | T3 N+ | E1309fs | R196* + W91* |  |  |  |  |  |  |  |
|  | **CLM 2** | T3 N+ | Q1367* | C277F |  |  |  |  |  |  |  |
|  | **EOC 11** | T4 N+ | R876* + E1309Dfs* | R248Q |  |  |  |  |  |  |  |
|  | **EOC 18** | T4 N0 |  | R249S |  |  |  |  |  |  |  |
|  | **CLM 3** | T3 N+ | E1286* | C176F |  |  |  |  |  |  |  |
| W&W | **EOC 19** | T3 N+ | / | | | | | | | | |

Legend: TRG, Tumor regression grade; W&W, Watch and Wait Approach.

**Supplementary Table 2: Mutations detected in tumor resection tissue samples.**

| **TRG** | **Patient ID** | **Final TNM Stage** | **Mutations of Resection** | | | | | | | | |
| --- | --- | --- | --- | --- | --- | --- | --- | --- | --- | --- | --- |
|  |  |  | **APC** | **TP53** | **EGFR** | **KRAS** | **NRAS** | **PIK3CA** | **SMAD4** | **AKT1** | **Other genes** |
| 1 | **EOC 5** | T0 N0 (0/13) |  |  |  |  |  |  |  |  |  |
|  | **EOC 6** | T0 N0 (0/14) |  |  |  |  |  |  |  |  |  |
|  | **EOC 13** | T0 N0 (0/18) |  |  |  |  |  |  |  |  |  |
|  | **EOC 14** | T0 N0 (0/16) |  |  |  |  |  |  |  |  |  |
| 2 | **CLM 1** | T3 N0 (0/12) | E1379* | V272M |  | G12V |  |  |  |  |  |
|  | **EOC 3** | T1 N0 (0/13) | P1453Lfs*20 | G244D |  | G13D |  |  |  |  | FBXW7: R505C |
|  | **EOC 9** | T3 N0 (0/18) |  |  |  |  |  |  |  |  |  |
|  | **EOC 16** | T3 N0 (0/15) |  | R273H |  | G12V |  |  |  |  |  |
| 3 | **EOC 10** | T3 N0 (0/24) | R1114* | R273C |  |  |  |  |  |  | MET: R988C |
|  | **EOC 4** | T2 N0 (0/13) |  | V216M |  |  |  |  |  |  |  |
|  | **EOC 12** | T3 N1 (2/26) | E1309Dfs* | Q100R |  | A146V |  |  |  |  |  |
|  | **EOC 15** | T2 N1 (1/16) |  | R213* |  |  |  |  |  |  |  |
|  | **EOC 17** | T2 N1 (2/20) | E1097* |  |  |  |  |  |  |  |  |
|  | **EOC 20** | T2 N0 (0/13) |  | I195T |  |  |  |  |  |  |  |
|  | **EOC 21** | T2 N0 (0/9) |  | R248Q |  |  |  |  |  |  |  |
|  | **CLM 4** | T3 N0 (0/12) |  | C238Y |  | G13D |  |  |  |  |  |
| 4 | **EOC 1** | T3 N0 (0/18) | Q1378* | S241F |  | A146V |  |  |  |  |  |
|  | **EOC 2** | T4 N0 (0/15) | R1114* + T1556Nfs*3 | Y220C |  | G12C |  |  |  |  |  |
|  | **EOC 7** | T3 N2 (7/30) | Q1367* (49.7%) | R213* (47.6%) |  |  |  |  |  |  | FBXW7: S582L |
|  | **EOC 8** | T3 N1 (2/18) | E1309fs | R196* + W91* |  |  |  |  |  |  |  |
|  | **CLM 2** | T3 N0 (0/18) | Q1367* | C277F |  |  |  |  |  |  |  |
|  | **EOC 11** | T4 N0 (0/11) | R876* + E1309Dfs* | R248Q |  |  |  |  |  |  |  |
|  | **EOC 18** | T3 N2 (6/21) |  | R249S |  |  |  |  |  |  |  |
|  | **CLM 3** | T2 N0 (0/12) | E1286* | C176F |  |  |  |  |  |  |  |
| W&W | **EOC 19** | / | / | | | | | | | | |

Legend: TRG, Tumor regression grade; W&W, Watch and Wait Approach.

**Supplementary Table 3: ctDNA yields in plasma samples**

| **TRG** | **Patient ID** | **ctDNA (ng/ml of plasma)** | | | | | | |
| --- | --- | --- | --- | --- | --- | --- | --- | --- |
|  |  | **T0** | **Tend** | **T4** | **T7** | **Top** | **TIMV** | **Tpost-op** |
| 1 | **EOC 5** | 9.97 | 6.30 | 13.23 | 10.84 | 12.41 | 13.12 | 51.75 |
|  | **EOC 6** | 7.27 | 7.87 | NA | NA | 4.50 | 11.55 | 31.91 |
|  | **EOC 13** | 7.42 | 4.35 | 5.66 | 6.00 | 4.80 | 4.91 | 19.72 |
|  | **EOC 14** | 7.95 | 8.51 | 9.78 | 5.14 | 9.97 | 12.04 | 10.42 |
| 2 | **CLM 1** | 6.19 | 5.81 | 6.26 | 6.22 | 5.25 | 6.22 | 14.06 |
|  | **EOC 3** | 5.51 | 5.58 | 6.49 | 5.21 | 12.33 | 15.41 | 49.87 |
|  | **EOC 9** | 7.01 | 4.54 | 6.41 | NA | 7.35 | NA | 27.22 |
|  | **EOC 16** | 13.05 | 26.81 | 17.62 | 9.71 | 16.12 | NA | 39 |
| 3 | **EOC 10** | 5.17 | 9.79 | 11.81 | 6.26 | 17.73 | 7.42 | 11.74 |
|  | **EOC 4** | 17.02 | 23.62 | 23.29 | 18.04 | 44.62 | NA | 51.07 |
|  | **EOC 12** | 11.1 | 18.86 | 9.04 | 9.71 | 19.09 | 18.86 | 54 |
|  | **EOC 15** | 4.69 | 13.65 | 12.04 | 10.13 | 20.70 | 20.66 | 13.65 |
|  | **EOC 17** | 6.49 | 8.48 | 4.95 | 6.86 | 11.14 | 28.43 | 11.59 |
|  | **EOC 20** | 4.65 | 6.83 | 7.84 | 6.49 | 25.05 | 21.11 | 37.16 |
|  | **EOC 21** | 4.43 | 6.53 | 4.16 | 6.94 | 12.41 | NA | 33.83 |
|  | **CLM 4** | 13.99 | 33.41 | 4.13 | NA | 25.54 | NA | 50.63 |
| 4 | **EOC 1** | 13.01 | 9.90 | 21.26 | 10.76 | 13.20 | 37.31 | 19.13 |
|  | **EOC 2** | 9.94 | 16.35 | 27.90 | 10.65 | 7.54 | NA | 9.90 |
|  | **EOC 7** | 6.45 | 12.79 | 8.89 | 5.51 | 11.74 | 9.90 | 48.00 |
|  | **EOC 8** | 7.16 | 9.00 | 4.88 | 7.05 | 11.14 | 16.05 | 37.54 |
|  | **CLM 2** | 5.29 | 4.54 | 4.31 | 8.55 | 6.98 | 7.05 | 10.20 |
|  | **EOC 11** | 5.63 | 5.33 | 6.56 | 6.79 | 8.29 | 10.95 | 42.75 |
|  | **EOC 18** | 3.83 | 4.16 | 5.59 | 7.46 | 42.75 | 68.25 | 18.60 |
|  | **CLM 3** | 7.16 | 4.46 | 6.34 | 7.39 | 11.25 | 113.63 | NA |
| W&W | **EOC 19** | / | | | | | | |

Legend: NA, plasma sample not available; TRG, Tumor regression grade; W&W, Watch and Wait Approach.

**Supplementary Table 4: Mutations and relative VAF detected in ctDNA from plasma samples**

| **TRG** | **Patient ID** | **Mutations detected in Blood Sample** | | | | | | |
| --- | --- | --- | --- | --- | --- | --- | --- | --- |
|  |  | **T0** | **Tend** | **T4** | **T7** | **Top** | **TIMV** | **Tpost-op** |
| 1 | **EOC 5** | **PIK3CA:**  E545K (0.15%) |  |  |  |  |  |  |
|  | **EOC 6** |  |  | NA | NA |  |  |  |
|  | **EOC 13** | **NRAS:**  Q61R (0.06%%) |  |  |  |  |  |  |
|  | **EOC 14** | **APC:**  Y1376Cfs*9 (0.33%);  **TP53:**  R282W (0.19%);  **NRAS:**  Q61L (0.1%);  **SMAD4:**  R361S (0.11%);  **GNAS:**  R201H (0.62%) | **GNAS:**  R201H(0.50%) | **GNAS:**  R201H (0.21%) | **GNAS:**  R201H (0.30%) | **GNAS:**  R201H (0.16%) | **GNAS:**  R201H (0.22%) | **GNAS:**  R201H (0.16%) |
| 2 | **CLM 1** | **APC:**  E1379*(0.22%) |  |  |  |  | NE |  |
|  | **EOC 3** | **KRAS:**  G13D (0.03%) |  |  |  |  |  |  |
|  | **EOC 9** | **KRAS:**  G12V (0.68%) |  |  | NA |  | NA |  |
|  | **EOC 16** | **TP53:**  R273H (0.40%);  **KRAS:**  G12V (0.50%) |  |  |  | **TP53:**  R273H (0.02%); | NA |  |
| 3 | **EOC 10** | **APC:**  R1114* (0.67%)  **TP53:**  R273C (0.48%) | **APC:**  R1114(0.04%) |  |  |  |  | **TP53:**  R273C (0.04%) |
|  | **EOC 4** |  |  |  |  |  | NA |  |
|  | **EOC 12** | **APC:**  E1309Dfs* (0.10%) |  |  |  |  |  |  |
|  | **EOC 15** | NE |  |  |  |  |  |  |
|  | **EOC 17** |  |  |  |  |  |  |  |
|  | **EOC 20** | **TP53:**  I195T (0.19%) |  |  |  |  |  |  |
|  | **EOC 21** | **TP53:**  R248Q (0.29%) |  |  |  |  | NA |  |
|  | **CLM 4** | **TP53:**  C238Y (0.01%) | NE |  | NA |  | NA |  |
| 4 | **EOC 1** | **APC:**  Q1378* (0.096%);  **TP53:**  S241F (0.06%) |  |  |  |  |  |  |
|  | **EOC 2** | **TP53:**  Y220C (0.78%)  **KRAS:**  G12C (0.29% ) |  |  |  |  | NA |  |
|  | **EOC 7** | **APC:**  Q1367* (1.20%)  **TP53:**  R213*(2.28%)  **FBXW7:**  S582L (1.75%) |  | **APC:**  Q1367* (0.22%)  **TP53:**  R213*(0.22%)  **FBXW7:**  S582L (0.13%): | **APC:**  Q1367* (0.22%)  **TP53:**  R213*(0.47%)  **FBXW7:**  S582L (0.04%): | **APC:**  Q1367* (0.26%)  **TP53:**  R213*(0.23%)  **FBXW7:**  S582L (0.80%): | **TP53:**  R213*(0.34%)  **FBXW7:**  S582L (0.30%): | **APC:**  Q1367*(0.04%)  **FBXW7:**  S582L (0.02%): |
|  | **EOC 8** | **APC:**  E1309fs (0.62%)  **TP53:**  R196* (0.18%) |  |  |  |  |  |  |
|  | **CLM 2** | **APC:**  Q1367* (0.16%)  **TP53:**  C277F (0.23%) |  |  |  | **APC:**  Q1367* (0.04%) |  |  |
|  | **EOC 11** | **APC:**  R876*(0.05%)  E1309Dfs* (0.22%)  **TP53:**  R248Q (0.06%) |  | **APC:**  E1309Dfs(0.03%) |  | **APC:**  R876*(0.02%) E1309Dfs*(0.1%) |  | **TP53:**  R248Q (0.01%) |
|  | **EOC 18** | **APC:**  E1408* (4.78%)  **TP53:**  R249S (4.92%) |  | **APC:**  E1408* (5.67%)  **TP53:**  R249S (6.08%) | **APC:**  E1408* (7.06%)  **TP53:**  R249S (7.08%) | **APC:**  E1408* (0.34%)  **TP53:**  R249S (0.35%) | **APC:**  E1408* (0.74%)  **TP53:**  R249S (0.50%) | **APC:**  E1408* (1.43%)  **TP53:**  R249S (2.02%) |
|  | **CLM 3** | **TP53:**  C176F (0.86%) |  |  |  | **TP53:**  C176F (0.01%) | NE | NA |
| W&W | **EOC 19** | / | | | | | | |

Legend: NA, plasma sample not available; NE, not evaluable; TRG; Tumor regression grade; W&W, Watch and Wait Approach.
